# Comments on ‘Insight into the history and trends of surgical simulation training in education: a bibliometric analysis’

**DOI:** 10.1097/JS9.0000000000000547

**Published:** 2023-06-16

**Authors:** Yongbin He, Haifeng Tang, Haiyang Wu, Guoxin Ni

**Affiliations:** a School of Sport Medicine and Rehabilitation, Beijing Sport University, Beijing; b Department of Spine Surgery, Graduate School of Tianjin Medical University, Tianjin; c Department of Rehabilitation Medicine, The First Affiliated Hospital of Xiamen University, Xiamen, China; d Duke Molecular Physiology Institute, Duke University School of Medicine, Durham, North Carolina, USA

*Dear Editor,*

Recently a paper in *Int J Surg* entitled ‘Insight into the history and trends of surgical simulation training in education: A bibliometric analysis’^1^ was published. We highly appreciate the efforts made by the author in this field. However, there are some issues that this research has we need to point out.

The authors mentioned in Section 2.1. Data collection and extraction that ‘Related articles from 1991 to 2020 were searched in the Web of Science (core collection database)’. Web of Science Core Collection (WSCC) includes: Science Citation Index Expanded (SCIE)–1900-present; Social Sciences Citation Index (SSCI)–1900-present; Arts and Humanities Citation Index (AHCI)–1975-present; Conference Proceedings Citation Index-Science (CPCI-S)–1990-present; Conference Proceedings Citation Index-Social Sciences and Humanities (CPCI-SSH)–1990-present; Book Citation Index-Science (BKCI-S)–2005-present; Book Citation Index-Social Sciences and Humanities (BKCI-SSH)–2005-present; Emerging Sources Citation Index (ESCI)–2018-present; Current Chemical Reactions (CCR-EXPANDED)–1985-present; Index Chemicus (IC)–1993-present. WSCC is primarily intended for researchers seeking published literature rather than bibliometric studies^2^. It is not suitable to utilize all these diverse databases with varying types and levels. For instance, ESCI serves as a valuable addition to the highly selective indexes by offering early visibility for sources undergoing evaluation as part of the rigorous journal selection process of SCIE, SSCI, and A&HCI^3^. More scholars are likely to choose to use the SCIE database for the sake of accuracy^4,5^.

In the same section, the strategy used during the search was (Supplementary data): #1=(((((TS=(teach*)) OR TS=(train*)) OR TS=(education)) OR TS=(practice)) AND TS=(surgery)) AND TS=(simulator); #2=(((((TS=(teach*)) OR TS=(train*)) OR TS=(education)) OR TS=(practice)) AND TS=(surgery)) AND TS=(simulation); #1 OR #2 (publication date to 2020-12-31) (7,014); (#1 OR #2) AND AK=(robotic) (publication date from 2000-01-01 to 2022-05-15) (443). The authors only used ‘surgery’ as search keywords, which may omit some useful articles. Besides, using ‘TS’ as a retrieval was likely to bring some irrelevant articles, such as ‘Cost-effectiveness of Total Knee Arthroplasty in the United States Patient Risk and Hospital Volume’ (Range NO.11 in the highest citations of author’s research) to the research topic ‘surgical simulation training in education’^6^. Using the database directly without bibliometric treatment is not suitable. Besides, the authors mentioned ‘After removing reviews, meeting reports, and letters, a total of 5258 papers were retrieved’. As far as we know, most researchers will only choose the type of articles for further analysis, other than reviews, meeting reports, and letters, there are proceeding paper, editorial material, book chapters, etc. It is better to choose the right type or to describe accurately.

The new strategy we used was ‘TI=((((teach*) OR (train*) OR education OR practice)) AND (surgery OR surgical OR surgeon* OR orthopedic* OR orthopedic* OR neurosurg* OR urologist*) AND (simulator OR simulation)) OR AB=((((teach*) OR (train*) OR education OR practice)) AND (surgery OR surgical OR surgeon* OR orthopedic* OR orthopedic* OR neurosurg* OR urologist*) AND (simulator OR simulation)) OR AK=((((teach*) OR (train*) OR education OR practice)) AND (surgery OR surgical OR surgeon* OR orthopedic* OR orthopedic* OR neurosurg* OR urologist*) AND (simulator OR simulation))’ within the publication year with a limit of 1991–2020, then only choose the type of articles. A total of 5714 and 4863 articles were published in the SCIE of WSCC, respectively. A big difference was found from the 7014 and 5258 articles in the original paper. Using the new strategy, we exclude the above-mentioned irrelevant articles but get more relevant articles such as ‘Virtual reality training improves operating room performance - Results of a randomized, double-blinded study’ (Range NO.1 in the highest citations of the research, the author’s research result missed this article. Supplementary Figure 1, Supplemental Digital Content 1, http://links.lww.com/JS9/A716). The keyword ‘robotic’ was added to the secondary searching within the publication date from 1 January 2000, to 15 May 2022. Finally, we got 261 relative articles can use for further analyze, it is still a big difference from the 348 articles in the original paper (Supplementary Figure 2, Supplemental Digital Content 2, http://links.lww.com/JS9/A717).

Engaging in discussions regarding issues in publishing papers is an integral part of research. This practice facilitates research improvement and brings it closer to the truth. It has been emphasized that authors bear the responsibility of employing accurate methods in their publications, while reviewers are accountable for identifying errors. Furthermore, readers also have the opportunity to highlight such problems in published articles. Chen *et al.* ^1^ published ‘Insight into the history and trends of surgical simulation training in education: A bibliometric analysis’ using inappropriate database, search strategy and search keywords. This could potentially mislead journal readers.

## Ethical approval

This study does not include any individual-level data and thus does not require any ethical approval.

## Author contribution

Y.H.: design, literature search, writing-original draft, collecting the data, writing- review and editing; H.T.: design, literature search, writing-original draft, collecting the data, writing- review and editing; H.W.: design, writing- review and editing; G.N.: design, writing- review and editing.

## Conflicts of interest disclosure

The authors declare no conflict of interest.

## Research registration unique identifying number (UIN)

1. Name of the registry: NA.
2. Unique Identifying number or registration ID: NA.
3. Hyperlink to your specific registration (must be publicly accessible and will be checked): NA.

## Guarantor

Guoxin Ni.

## Supplementary Material

SUPPLEMENTARY MATERIAL
