# Supplementary figures and images for: Comments on ‘Insight into the history and trends of surgical simulation training in education: a bibliometric analysis’
Source: Int J Surg. 2023 Jun 16;109(10):3228–9. doi: 10.1097/JS9.0000000000000547 (PMC10583962; doi:10.1097/JS9.0000000000000547)

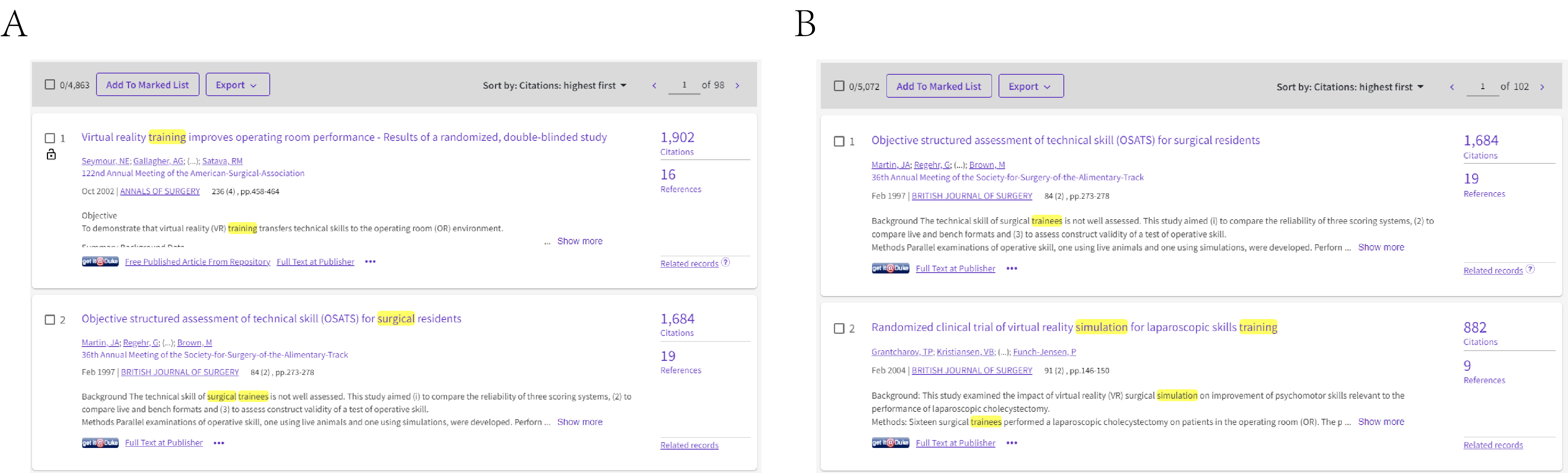

Supplement: SUPPLEMENTARY MATERIAL [file js9-109-3228-s001.jpg]

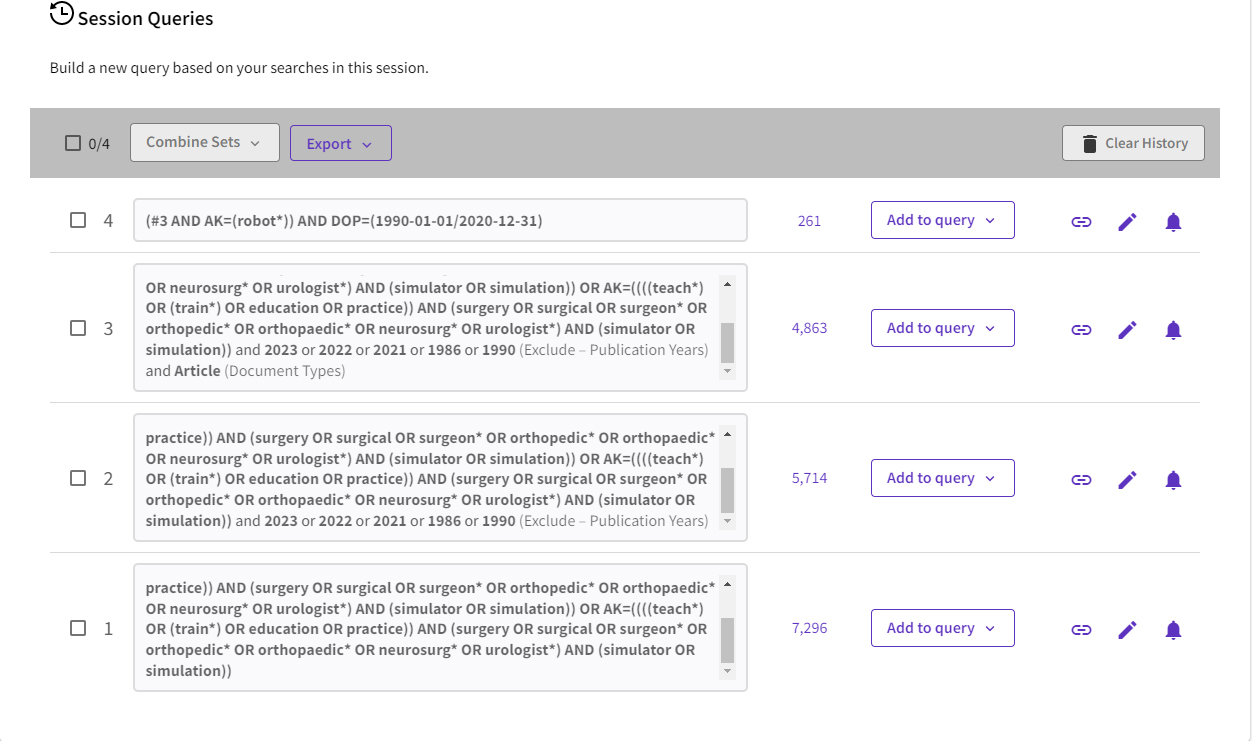

Supplement: SUPPLEMENTARY MATERIAL [file js9-109-3228-s002.jpg]
